# Assessing the Association Between Pakistani Women’s Religious Beliefs and Sports Participation

**DOI:** 10.3389/fpsyg.2022.915521

**Published:** 2022-06-10

**Authors:** Rizwan Ahmed Laar, Muhammad Azeem Ashraf, Shu Zhou, Lei Zhang, Zhengliang Zhong

**Affiliations:** ^1^College of Physical Education, Hubei Normal University, Huangshi, China; ^2^Research Institute of Educational Science, Hunan University, Changsha, China; ^3^School of Marxism, Hubei Normal University, Huangshi, China

**Keywords:** religious beliefs, sports participation, Pakistan, women sport, SCSRFQ

## Abstract

Women’s participation in physical activities has been discouraged for a variety of reasons, especially in Muslim countries. This study aims to highlight Pakistani women’s religious beliefs about sports. It focuses on whether their religion contradicts their participation in sporting activities, and it does so by using an adapted version of the Santa Clara Strength of Religious Faith Questionnaire (SCSRFQ) in the theoretical context of feminism in sports. The snowball sampling method was used to select women (*n* = 357) from the Sindh province of Pakistan, who completed a questionnaire incorporating the SCSRFQ that was specially designed for the current study. The results were unexpected, revealing that religious beliefs have no negative impact on Pakistani women’s participation in sports. Few participants (14 out of 357, 3.9%) believe that religion is an obstacle to their participation in sports. The results of the study challenge the traditional view by most of the previous studies that Islam is against women’s sports. It also challenges cultural limitations, such as some unwritten rules in Pakistani culture according to which women cannot participate in sports. These efforts should lead to enhanced female sports participation in the Pakistani context.

## Background of the Study

Taking part in sports is helpful for the vitality of the body and mind. “A sound mind in a sound body” is a common saying. Regular participation in physical activity and exercise is beneficial for a variety of physiological systems and improves people’s quality of life (Lim and Taylor, 2005; Forkan et al., 2006; King et al., 2007). It is recommended that adults engage in an average of 30 min of moderate-intensity activity daily to maintain health and prevent chronic disease (Nelson et al., 2007). For senior citizens, it is recommended that individuals engage in moderate physical activity with an average of 3–6 metabolic equivalent tasks (METs) daily, the equivalent of walking fast at 3–4 mph (Patte et al., 1995). Despite such suggestions, many people prefer to live a more sedentary lifestyle for many reasons (Manaf, 2013).

Pakistan began to establish sports support federations in the year following its independence in 1947. In 1948 alone, the Pakistan Cricket Board (PCB), Pakistan Hockey Federation (PHF), Pakistan Tennis Federation (PTF), Pakistan Tenpin Bowling Federation (PTBF), and Pakistan Swimming Federation (PSF) were established. At present, 40 national sports federations are affiliated with the ministry and the board [that is, the Inter Provincial Coordination (IPC) and the Pakistan Sports Board (PSB)]. Each federation is responsible for the promotion and development of its own games/sports; some function well, while others need to be improved in many ways. Despite these supportive federations, the participation and performance of Pakistani athletes, especially women, in the national and international arena need to enhance right away. Almost 90% of women and girls do not participate in sports or physical activities (Cailliau, 2013; Laar et al., 2019a). Although Pakistan’s female athletes have participated in the summer Olympics since 1996 (six summer Olympics), they have won no medals (Laar, 2019).

This quantitative study describes the religious beliefs of Pakistani women, focusing on whether their beliefs inhibit their participation in physical activities (PAs). It does so by using an adapted version of the Santa Clara Strength of Religious Faith Questionnaire (SCSRFQ) and through the theoretical lens of feminism in sports. This study adds to the literature examining women’s levels of participation in sports in Muslim countries and the influence of religious beliefs on those levels. It specifically asks whether religious beliefs impact Pakistani women’s participation in sports since there is a gap in the literature related to assessing Pakistani women’s sports participation and their religious beliefs, and in particular, it answers this question with the help of the SCSRFQ (Coakley and Pike, 2014). This study supports much of the previous sports literature and is consistent with the findings from other Muslim countries. This comparison enables us to become some of the first researchers in this field. While a few books and articles have been written from this perspective, especially regarding the validity of the SCSRFQ (see Sherman et al., 2001; Plante, 2010; Dianni et al., 2014; Pakpour et al., 2014) and women’s participation in Muslim countries, the list is by no means exhaustive, and the existing literature may oversimplify the relationship between religious faith and female sports participation by using the SCSRFQ in Muslim countries, especially Pakistan (Laar, 2019; Laar et al., 2019a,b, 2020). However, it is also important to study the religious faith of women by using an adapted version of the SCSRFQ in a Muslim country (in this case, Pakistan) to explore whether such faith contradicts sports participation. This research has the potential to open a new understanding of the links between women’s religious beliefs and their level of sports participation and to serve as a basis for constructive suggestions regarding the development of women’s sports in Pakistan.

### Religion and Sports in Islam and the Interpretation of This Relationship in the Pakistani Context

In Pakistan, religion has a greater influence on most aspects of people’s lives than in other Islamic countries (Ida and Saud, 2020) since more than 90% of the population is Muslim (Ashraf, 2018; Laar et al., 2019a). An understanding of the basics of Islam is therefore important for this study. Islam, from the Arabic root word “Salam,” which means “peace,” is a faith held by one-fifth of the world’s population. Muslims believe in one Allah (God) who revealed the holy Quran to Muhammad, the final prophet, ~1,400 years ago. According to Kahan (2003), in Islam, activities of daily life are governed by the Quran and the Hadith (Mohammed’s maxims and practices). Islam prohibits dishonesty, gambling, murder, suicide, bribery, theft, forgery, interest, the consumption of alcohol and pork, hoarding, cruelty to animals, public nudity, and adultery. Instead, Islam promotes tolerance for non-Muslims, eating clean food, respect for parents and family units, generosity, marriage and a stable family life, assistance for poor individuals, the decent treatment of women, and engagement in healthy recreation (Kahan, 2003).

Regarding sports in Islam, horse riding, swimming, shooting, hunting, fencing, wrestling, and running are referred to in the Quran and the Hadith (Kahan, 2003). It is well known that women take part in wars and activities to obtain ‘an adequate level of physical health and military trainings’ (Kahan, 2003). Mohammed’s 55-year-old wife, Khadijah, is said to have climbed mountains to provide food to her husband (Kahan, 2003). The original form of Islam was inherently concerned with the development and maintenance of the spirit and physical strength, regardless of gender (De Knop et al., 1996; Adamczyk and Felson, 2012; Khan et al., 2020). However, previous studies of religious beliefs about sports in the Pakistani context reveal that modern Muslim women’s participation in physical activities is subject to religious and cultural constraints (Fazal et al., 2019), the ethos of college physical education (PE) and sports facilities (Laar et al., 2019b). Despite these constraints, Muslim women in Pakistan show a positive attitude toward sports, and religion is considered less of a constraint than other social and economic factors by the respondents of the study conducted by Laar et al. (2019b). However, very limited research is available about the issues that influence women’s participation in physical activities in the Pakistani context (Nanayakkara, 2012; Laar et al., 2019a).

In Pakistan, the interpretation of Islam is mainly reserved for religious scholars, i.e., imams (officiating priests of a mosque), rather than people directly referring to the Quran themselves. In many cases, the Quran is misinterpreted, creating serious consequences in many fields, including women’s outdoor activities and sports. There are some unwritten rules that are followed; for example, women cannot participate in sporting activities. Women’s participation in sports is often viewed as bad in religious terms. However, as discussed above, the Quran promotes high-quality health and encourages men and women to participate in PE to maintain a healthy lifestyle (Qureshi and Ghouri, 2011). As Miles and Benn (2016) point out, it is not the religion that is against sporting activities; rather, ‘the cultural requirements of the western and Islamic sports related environment conflicts with each other.’

### Scenario of PE (as an Academic Subject) in Pakistan

If someone wants to understand the value of physical activity in any society, a consideration of PE in the society’s schooling system is very important. PE is an educational process that uses physical activities as a resource to help individuals acquire skills, health, knowledge, and attitudes that contribute to their best development and well-being. As mentioned above, physical activity on a daily basis during childhood and adolescence is very important for well-being and the prevention of various health conditions. According to Fatima (2019), the school environment is one of the most effective zones for planning and implementing interventions to encourage PE. However, there is not a single university/college in Pakistan fully dedicated to PE, although there are some universities and colleges that offer PE as a subject. Games (cricket, badminton, football, hockey, squash, snooker, and traditional games such as Kabaddi “wrestling”) and athletics are the main activities in Pakistani institutions (Kamal and Khan, 2014).

In Pakistan, sports have not been given the attention they deserve (Fatima, 2011), and for some, they are still considered an extracurricular activity or hobby. Sports science is a vast field, but in Pakistan, it is not been given the actual meaning (Yasmeen, 1997). According to Fatima (2011), in Pakistan, the subject is considered a waste of time, and people who choose it are ridiculed. There is a lack of understanding of the importance, necessity, and scope of PE in Pakistan, despite its potential positive contribution to many social issues. Since a healthy body has a healthy mind, which actually helps to stay healthy (Kamal and Khan, 2014), sports have the potential to improve psychological behavior. This paper begins with an overview of sports and Islam in general and in the Pakistani context in particular. The focus then shifts to the current situation of PE as an academic subject in Pakistan. This is followed by a review of the relevant literature that has used the SCSRFQ, and then, the methodology and findings are described.

### Santa Clara Strength of Religious Faith Questionnaire

According to Fetzer Institute/National Institute on Aging Working Group (1999), Sherman et al. (2001), and Hill et al. (2007), both empirical research and clinical practice need to use reliable, effective, and practical tools to evaluate spiritual and religious behavior, ideas, and practice. Sherman et al. (2001) and Hill et al. (2007) state that with the development of research in recent years, there are a large number of self-report instruments on behaviors, practices, and spiritual and religious beliefs. However, many of these tools are very lengthy and unsuitable for specific research environments, while others have little empirical research to support their reliability and validity. The SCSRFQ was developed and published in 1997 in the *Journal of Pastoral Psychology* and has since been reprinted several times (Avants et al., 2003; Batson and Shwalb, 2006; Plante, 2009). It is a short self-report measure using 10 items (or a short version of five items) to assess the intensity of and participation in religious beliefs. The original version of the scale consists of 10 items that use a four-point Likert scale from strongly disagree to strongly agree. The following are example items: “I pray daily,” “My religious faith is extremely important to me,” and “I look to my faith as a source of inspiration.” According to previous studies (Plante and Boccaccini, 1997, 1997a), the SCSRFQ assesses participants’ strength of religious faith regardless of their religious affiliation or denomination. It is applicable to people with no interest in or connection with religious organizations, traditions, and views (Plante and Boccaccini, 1997; Plante et al., 1999, 2002; Sherman et al., 1999, 2001; Storch et al., 2004). The SCSRFQ is valid and has also been found by Plante and Boccaccini (1997) to have high internal reliability (Cronbach’s alpha = 0.95) and split-half reliability (*r* = 0.92). In addition, the SCSRFQ is easy to obtain free of cost.

Studies of the internal consistency of the scale have found a correlation ranging from 0.94 to 0.97, whereas based on a split-half reliability score and Cronbach’s alpha score, the range is from 0.90 to 0.96 (Plante and Boccaccini, 1997; Plante et al., 1999, 2002; Sherman et al., 1999). The SCSRFQ is correlated with other quality religious faith instruments, such as the Age Universal Religious Orientation Scale (AUROS), *r* = 0.70 to 0.90, and the Religious Life Inventory (RLI), *r* = 0.76 to 0.90. The SCSRFQ is also correlated with the Duke Religious Index (DRI), *r* = 0.71 to 0.85 (Cronbach, 1951; Schmitt, 1996; Plante and Boccaccini, 1997; Plante et al., 1999, 2002; Sherman et al., 1999). Therefore, existing research shows that the SCSRFQ is a highly reliable instrument for measuring religious beliefs and their impact on other social aspects of life (Plante and Boccaccini, 1997; Hu and Bentler, 1999; Plante et al., 1999, 2002; Sherman et al., 1999). The main purpose of the current research is to highlight the religious beliefs of Pakistani women about sports by using an adapted version of the SCSRFQ based on the theoretical perspective of feminism in sports and to focus on whether their religious beliefs contradict their participation in sporting activities, while keeping the hypothesis that religious believes may impact the women sport participation and the age and marital status of the respondents have the different attitude towards sports participation.

## Methods

This study was conducted in the Muslim country of Pakistan in August 2019. It is part of a larger project investigating women’s participation in sports and physical activities and its influence on various factors, including religion, socioeconomics, culture, and family (see Laar, 2019; Laar et al., 2019a,b, 2020). All participants were female Suni Muslims and were selected from the Sindh province (2nd largest province of Pakistan) using snowball sampling (Laar et al., 2019b, 2021). As in previous research (Young et al., 2003; Mirsafian et al., 2014; Laar et al., 2019a), this study also conducted a survey to gain quantitative data. In the first stage, a sample of 400 participants was selected. The final sample size of valid responses, after data collection and using AMOS, was 357, yielding a response rate of 89.2% (Table 1). Notable, the sample was calculated with the help of Sample Size Calculator (SSC). The data collection process was completed in 5 weeks. The major characteristics of the sample (*n* = 357) are as follows: age—16–19 (*n* = 60, 16.8%), 20–23 (*n* = 155, 43.4%), and 23 and above (*n* = 142, 39.8%); participating in sport (*n* = 186, 52.1%); education—intermediate (*n* = 52, 14.5%), bachelor’s (*n* = 122, 34.2%), master’s (*n* = 157, 44%), and PhD (*n* = 26, 7.3%); school campus—girls’ campus (*n* = 118, 33.1%) and coeducation (*n* = 239, 66.9%); marital status—unmarried (*n* = 257, 72%) and married (*n* = 100, 28%); and school type—public (*n* = 267, 74.8%) and private (*n* = 90, 25.2%; see Table 1).

**Table 1:** Background information of the participants.

Elements	Frequency	Percentage
**Sample distribution**		
Total female students	400	100
Valid responses (after data collection and using AMOS)	357	89.2
Participating in sport	186	52.1
**Age (Mean = 2.23 ± SD = 0.71)**		
16–19 years	60	16.8
20–23 years	155	43.4
Over 23 years	142	39.8
**Education**		
Intermediate	52	14.5
Bachelor’s	122	34.2
Master’s	157	44.0
PhD	26	7.3
**School campus**		
Girls’ campus	118	33.1
Coeducation	239	66.9
**Marital Status**		
Unmarried	257	72.0
Married	100	28.0
**School type**		
Public	267	74.8
Private	90	25.2

Based on the aim of the study, a two-part questionnaire was created based on the previous literature (Qureshi and Ghouri, 2011; Mirsafian, 2014; Mirsafian et al., 2014; Zvan et al., 2017; Laar et al., 2019a). The first part requested background- and sports-related information from the participants; the second part addressed their Islamic beliefs using the SCSRFQ, which is based on a Likert scale (Jackson, 1983; Henderson et al., 1988; Sherman et al., 2001; Plante, 2010; Dianni et al., 2014; Laar et al., 2019a) ranging from (1) strongly disagree to (4) strongly agree. The questionnaire was designed in the English language, which is the second most commonly spoken language and the official language of Pakistan. In the current study, the standard procedure for developing and establishing the psychometric characteristics of the scale was followed. These measures are generally designed for scales, especially for the development of religious and/or spiritual (R/S) scales. The authors acknowledge that they are not scale psychometrics or translation experts (readers may wish to review Boateng et al., 2018, and Koenig and Al Zaben, 2021 for a more in-depth description and definition).

Before the survey, the participants were informed of the purpose of the current research and that participation was voluntary. Their affiliations and identity are not revealed to ensure the confidentiality and privacy of their data. Current research provides opportunities to understand the current status of women’s participation in sports in Pakistan and whether their religious beliefs are inconsistent with their participation in sports. It is worth noting that these types of data (especially about women) are often difficult to obtain in Pakistani society, making it extremely important and valuable; thus, confidentiality was essential. Notably, a one-sample *t*-test, independent sample *t*-test and multivariate analysis (MANOVA) were used to analyze the data. The results of the survey then underwent a Pearson correlation test, with the primary focus on the following question: does the strength of their religious beliefs influence the sports participation of Pakistani women?

### Statistical Analyses

First, normality test was conducted to ensure the data is approximately normally distributed, followed by one-sample *t*-test of the SCSRFQ items to determine the participants’ religious beliefs. All the items were analyzed through test variable(s). On the other hand, to analyze the participants’ level of participation in sports, *t*-tests were applied to sports participation items and to the unmarried and married variables. “Marital status” was assigned as “grouping variable,” whereas the rest of the items were categorized as “test variables” (see Table 3). Moreover, multiple regression model including marital status and age as predictors, with attitudes toward sports participation as the dependent variable was conducted to examine the relationship of the variances (see Table 4). In addition, to analyze the question “do religious beliefs affect participants” participation in sports?, the interrelations among the items (questions) were measured, and bivariate correlation analyses were run to obtain the results (see Table 5). Notably, a one-factor model was postulated. The models were tested using confirmatory maximum likelihood (ML) factor analysis parameter estimates in AMOS 24. Data were filtered by eliminating all defective samples.

## Findings

The aim of the study was to assess the impact of religious beliefs on sports participation using a questionnaire incorporating the SCSRFQ. Notably, skewness and kurtosis *z*-values of data is in between −1.96 and +1.96 and the Shapiro–Wilk test value of *p* is above 0.05 (*p* > 0.05) in most of the cases. Hence, example data of this study are a little skewed and kurtotic, but it does not differ significantly from normality. It can be assumed that data are approximately normally distributed, in terms of skewness and kurtosis. The results reflect three steps: measuring religious beliefs and sports participation, conducting confirmatory factor analysis (CFA), and determining internal consistency reliability, which mainly pertain to the SCSRFQ (Sherman et al., 2001; Plante, 2010; Dianni et al., 2014; Pakpour et al., 2014). Descriptive statistics established that the mean strength of religious faith score, as assessed by the SCSRFQ, was 30.40 (SD = 8.10). Judging by the authors of the SCSRFQ questionnaire, this score seems to be relatively high while having alpha coefficient = 0.82.

### Measuring Religious Beliefs and Sports Participation

The main purpose of this study is to observe whether religious beliefs inhibit women’s sports participation in Pakistan. To answer the research question, the data were analyzed in three sub-steps.

#### Religious Beliefs

The results of the women who practice sports and those who do not regarding the SCSRFQ items shows that the responses of 6 item out of 10 are significantly different with a *p*-value of <0.05 (*p* < 0.01; see Table 2). Whereas, rest of the four items such as: Q14, Q19, Q20, Q22 show the similarity in responses (*p* > 0.05). In addition, Table 2 highlights the validity and factor loading of all items in both instruments with h^2^. The results indicate that factor analysis of both instruments, adapted to the SCSRFQ and other items (Table 4), was appropriate for the data (Stevens, 1996). Items with a factor loading of more than 0.40 were considered to have loaded onto one factor (Hinkin, 1995; Stevens, 2012).

**Table 2:** SCSRFQ items *t*-tests: Differences between sports participating and non-participating women with factor loadings and h^2^.

SCSRFQ items	Q8. Do you participate in sports?	Mean	±SD	*t*	Value of *p*	Factor Loadings	h^2^
Q13. My religion faith is extremely important to me.	Yes	3.35	0.69	3.438	0.001	0.67	0.75
	No	3.08	0.81				
Q14. I pray daily.	Yes	3.06	0.79	0.823	0.411	0.91	0.58
	No	2.99	0.84				
Q15. I look to my faith as a source of inspiration.	Yes	3.26	0.73	3.330	0.001	0.82	0.66
	No	2.99	0.77				
Q16. I look my faith as providing meaning and purpose in my life.	Yes	2.21	1.03	−1.676	0.095	0.76	0.71
	No	2.40	1.09				
Q17. I consider myself active in my faith.	Yes	3.06	0.77	−0.842	0.400	0.78	0.49
	No	3.13	0.79				
Q18. My faith is an important part of who I am as a person.	Yes	3.25	0.79	2.600	0.010	0.82	0.53
	No	3.02	0.84				
Q19. My relationship with God is extremely important to me.	Yes	3.40	0.79	1.057	0.291	0.59	0.65
	No	3.30	0.88				
Q20. I enjoy being around others who share my faith.	Yes	3.02	0.78	0.189	0.850	0.83	0.51
	No	3.00	0.83				
Q21. I look my faith as a source of comfort.	Yes	3.24	0.71	2.857	0.005	0.72	0.63
	No	3.02	0.77				
Q22. My faith impacts many of my decisions.	Yes	3.08	0.80	0.555	0.579	0.58	0.71
	No	3.03	0.77				

#### Sports Participation

Table 3 illustrated the type of sport generally participated by the female participants. The ratio of participating in Cricket is high followed by badminton 65.59% and 34.94%, respectively. Table 4 shows that married and unmarried participants did not demonstrate a large difference in their sports participation (Q8, “Do you participate in sports?”). This result reveals that compared with unmarried women, married women participate slightly more in sporting activities. For all the remaining sporting-related items, married and unmarried participants showed similar responses indicating that sports are important to them. However, some of women still did not participate for different reasons (Q7). On the other hand, both groups disagree that Islam restricts sports participation (Q9). The ratio of married to unmarried participants saying that Islam does not allow women to participate in PE is also almost the same (Q11.7), and the rate of married and unmarried women suggesting that controlling religious and cultural restrictions can enhance female sports participation in Pakistan also does not show a large difference (Q12.2; see Table 4). The validity and communalities of all items in both instruments with h^2^ are highlighted in Table 4.

**Table 3:** Sports played by Pakistani women.

Sport	n	%
Badminton	65	34.95
Cricket	122	65.59
Yoga	12	6.45
Table tennis	27	14.52
Gymnastic	18	9.68
Swimming	15	8.06
Shooting	11	5.91
Running	7	3.76
Aerobics/Dance	6	3.23
Basketball	12	6.45
Football	21	11.29
Volleyball	12	6.45
Tennis	32	17.20
Bike	45	24.19
Hockey	8	4.30

Only 186 female students are enabling to respond to this question rest of the female students do not participate in any sports.

**Table 4:** Sports participation item *t*-tests: Differences between unmarried and married participants with factor loadings and h^2^.

Dimension	Marital status	Mean	±SD	*t*	*Value of p*	Factor loadings	h^2^
Q7. Physical activity is important to me.	Unmarried	3.36	0.75	−1.302	0.194	0.69	0.69
	Married	3.47	0.56				
Q8. Do you participate in sports?	Unmarried	1.44	0.497	−2.637	0.009	0.81	0.58
	Married	1.59	0.494				
Q9. Do you think that, according to the teachings of Islam, males/females should not do sports?	Unmarried	2.17	1.039	0.010	0.992	0.58	0.73
	Married	2.17	0.965				
Q11.7. Religion does not allow	Unmarried	1.97	0.174	1.261	0.208	0.55	0.64
	Married	1.94	0.239				
Q12.2. Control religious and cultural restrictions	Unmarried	1.77	0.424	−0.271	0.787	0.69	0.77
	Married	1.78	0.416				

To examine the relationship between attitudes toward sports participation (dependent variable) and participant characteristics, such as marital status and age, multiple regression model was designed. The results show that the attitude of young and unmarried participants toward sports participation or importance is more positive (Table 5). Notably, the observed covariance matrices of the dependent variables are equal across groups (*p* > 0.05). The Age and Marital status significantly predicted only one dependent variable (attitudes toward sports participation Q8) *F* = 12.62, and *p* < 0.005 (Table 5). Whereas, there is no difference in responses based on age or marital status of the participants in rest of variables. Hence, author reject hypothesis which says there is significant difference between the age and marital status towards attitude of sports participation.

**Table 5:** Multiple regression model (age and Marital status as predictors with attitudes toward sports participation).

Regression weights		Beta coefficient	R2	F	*t*-value	Value of *p*	Sig. (Model)
Attitudes toward sports participation (Q7)	Age	0.03	0.06	1.03	0.60	0.546	0.36
	Marital status	0.10			1.18	0.241	
Attitudes toward sports participation (Q8)	Age	−0.15	0.06	12.62	−4.24	0.000	0.00
	Marital status	0.20			3.40	0.001	
Attitudes toward sports participation (Q9)	Age	−0.21	0.02	3.99	−2.82	0.005	0.02
	Marital status	0.06			0.48	0.630	
Attitudes toward sports participation (Q10)	Age	0.03	0.01	0.126	0.44	0.659	0.88
	Marital status	−0.03			−0.31	0.754	

Q7, Physical activity is important to me. Q8, Do you participate in sports? Q9, Do you think that, according to the teachings of Islam, males/females should not do sports? Q10, Participation in physical activities is mainly not encouraged by my own family members and by society and culture.

#### Correlation Between the Strength of Religious Beliefs and Sports Participation

Pearson product correlation of total score for the SCSRFQ and each of the sports participation items was found to be very low positive and negative correlation (*r* = 0.06, −0.13, −0.06, 0.03, respectively and *p* > 0.05 except the correlation of total score of SCSRFQ with Q8; see Table 6). This shows that there is no difference in responses regarding the attitudes toward sports participation based on age or marital status. In other words, it can be narrated that, religion believes of the participants of current study does not impact their attitudes towards sports participation.

**Table 6:** Pearson correlation of total score for the SCSRFQ with each of the sports participation items.

	Total score of SCSRFQ&Q7	Total score of SCSRFQ&Q8	Total score of SCSRFQ&Q9	Total score of SCSRFQ&Q10
Pearson correlation	0.06	−0.13^*^	−0.06	0.03
*Value of p*	0.21	0.02	0.22	0.55

* Correlation is significant at the 0.05 level (two-tailed). Q7, Physical activity is important to me. Q8, Do you participate in sports? Q9, Do you think that, according to the teachings of Islam, males/females should not do sports? Q10, Participation in physical activities is mainly not encouraged by my own family members and by society and culture.

## Discussion and Conclusion

This study provides a complete understanding of religious beliefs and the sports participation of Pakistani women, mainly focusing on the question of “do religious beliefs influence the participation of Pakistani women in sports?” According to the results, most participants have religious beliefs (with an average value of 3.05 out of 4), mainly Islam. They practice their faith regularly, and it influences many of their decisions in life. These findings match the results of other studies, such as Laar et al. (2019b) and Ashraf (2018), who argue that religion has a greater impact on most of the aspects of life in Pakistan than in other Islamic nations. In Pakistan, more than 90% of the population practices Islam. The results of this study reveal that although almost 50% of the participants do not participate in sporting activities, the majority believe that it is very important to participate in sporting activities and that Islam does not have any conflict with sports participation (Miles and Benn, 2016; Oates, 2016; Laar et al., 2019a,b). These results can be explained such as: in Pakistan, many people perform the cultural (including traditional sports) and ritual activities together which can normally see in national media too, which proofs the good image of religion and does not leave a conflict nature of religion with sports for the people of Pakistan. This study shows that the sports participation rate of unmarried women is higher than that of married women. These results may be explained by the tendency of men to restrict their wives’ outside activities. As Laar et al. (2019b) and Hakim and Aziz (1998) observe that, in Pakistan, patriarchy is widespread, with men mainly (but not exclusively) controlling their wives’ external activities.

The main objective of the current study is to assess the impact of religious beliefs on sports participation among Pakistani women from the theoretical perspective of feminism in sports and using the SCSRFQ. According to the results shown in Table 6, individuals’ religious beliefs do not have any negative impact on Pakistani women’s participation in sports. The correlation of the variables related to the religious believes and not practicing sports is significantly low. These results were unexpected and surprising (alternative hypothesis H_a_). Because much of the previous literature (e.g., Haley et al., 2001; Fitzgibbons, 2015) claims that religion can reduce sports participation, especially among women. These results make this study relevant and unique in the field of measuring the religious believes and sports participation especial in Pakistani women context. However, there are some exceptional studies that highlight that religion (mainly Islam) does not prevent women’s participation in sports (Miles and Benn, 2016; Oates, 2016; Laar et al., 2019b). In addition, Miles and Benn (2016) note that any reduction is not a consequence of religion *per se* but of the conflict between the cultural requirements of Islamic and Western sports-related environments, such as the wearing of sporting clothes and the intermingling of the sexes during sports participation. However, very few participants in this study (14 out of 357, 3.9%) thought religion was an obstacle to their sports participation, whereas 82 participants (23%) suggested that controlling religious and cultural limitations could enhance women’s sports participation. This study was based on women in only one province of Pakistan; thus, there might be a difference of opinion if men in Pakistani society are surveyed. However, our study focused only on women’s religious beliefs and the impact of their beliefs on their sports participation. The majority of the participants had religious beliefs, but according to the participants of this study, these religious beliefs did not reject women’s sports participation in Pakistan.

This study has many implications that could contribute to Pakistan in different ways. First, although the results of this study show that religious beliefs do not have any strong influence on Pakistani women’s sports participation, the levels in Pakistan are low (Klein, 2007; Mirsafian et al., 2014; Laar et al., 2019a,b, 2020). To tackle this problem, unwritten and misleading rules regarding Islam should be vigorously challenged: Islam is not an anti-sports religion (Benn and Ahmed, 2006; Benn et al., 2010). Second, according to Laar et al. (2019b), the media should represent images of real sportswomen who would surely impress many of their fans. This would be helpful in enhancing women’s sports participation in Pakistan. Finally, and most importantly, women themselves should learn from feminist theory that they should stand up for their rights in this male-dominated society. They should stand firm and not let themselves be oppressed by the tactics of other society members.

Discovering the underlying reasons for these unwritten and misleading rules could be a challenge for future studies with larger samples. However, these perceptions might be explained by the fact that, as a religion, Islam is open for individual interpretation. Pakistan has great talent in sports, but there is a lack of opportunities and facilities that hold the country back in this field (Laar et al., 2019b, 2020). If we can bring physical activities back to educational institutions, Pakistan can surely enhance sports participation and performance in sporting events, especially for women. Furthermore, an internal shift in the traditional view that Islam is against women’s sports and the corresponding cultural limitations should increase participation in sports and society. Programs demonstrating the benefits of sports for parents and women can also help in better understanding the topic. The reasons for the lack of participation of Pakistani athletes, especially in international events such as the Olympics, may represent another fascinating question for future research. Future research could also focus on using the SCSRFQ with more participants (especially interviewing husbands) and study populations. Sports educators who use the scale are encouraged to publish their findings in a journal.

### Study Limitations

This study has some limitations. First, the participants were female only, and the responses of male participants may be different. Second, the study was conducted in one province only; thus, it may not represent the entire population. Third, all participants belonged to one sector of Islam (Sunni Muslims), and the responses of the Shia sector of Islam may show different opinions. Fourth, asking female respondents whether religious beliefs inhibit women’s participation in sports may have caused some endogeneity issues. Fifth, the geographical, sociocultural, and religious elements of the selected population were similar, which may have led to bias. The investigators ensured that any bias and endogeneity issues were addressed to avoid such an outcome.

## Data Availability Statement

The raw data supporting the conclusions of this article will be made available by the authors, without undue reservation.

## Ethics Statement

The study was conducted in accordance with the Declaration of Helsinki, and the protocol was approved by the Ethics Committee of the School of Sports Science and Physical Education Hubei Normal University. We completely followed the relevant ethical considerations. The patients/participants provided their written informed consent to participate in this study.

## Author Contributions

RL: conceptualization, methodology, and writing-original draft preparation. MA, SZ, LZ, and ZZ: writing-review and editing. All authors contributed to the article and approved the submitted version.

## Conflict of Interest

The authors declare that the research was conducted in the absence of any commercial or financial relationships that could be construed as a potential conflict of interest.

## Publisher’s Note

All claims expressed in this article are solely those of the authors and do not necessarily represent those of their affiliated organizations, or those of the publisher, the editors and the reviewers. Any product that may be evaluated in this article, or claim that may be made by its manufacturer, is not guaranteed or endorsed by the publisher.
